# Long-Term Efficacy and Safety of a Low-Carbohydrate Diet in Type 2 Diabetes Remission: A Systematic Review

**DOI:** 10.7759/cureus.93340

**Published:** 2025-09-27

**Authors:** Rajib Das, Nour Mohammad, Md Shaiful Islam, Sowmitra Das, Faisal Abdullah, Md. Abdul Kader, Md Abdullah Al Mamun, Sourav Dutta, David Unwin, Istvan Mazak

**Affiliations:** 1 Epidemiology, University of Chester, Chester, GBR; 2 Endocrinology, South Warwickshire University NHS Foundation Trust, Warwick, GBR; 3 General Medicine, University Hospitals Dorset NHS Foundation Trust, Dorset, GBR; 4 General Medicine, Northern Care Alliance Foundation Trust, Salford, GBR; 5 General Medicine, Imperial College Healthcare NHS Trust, London, GBR; 6 Epidemiology and Public Health, North South University, Dhaka, BGD; 7 Biochemistry and Molecular Biology, Dhaka University, Dhaka, BGD; 8 Emergency Medicine, Maidstone and Tunbridge Wells NHS Trust, Maidstone, GBR; 9 Faculty of Health Social Care and Medicine, Edge Hill University, Ormskirk, GBR; 10 Renal Medicine, Croydon University Hospital, Croydon, GBR

**Keywords:** classic ketogenic diet, diabetes mellitus type 2, diabetes reversal, low-carbohydrate diet, type 2 diabetes remission, very low-calorie diet

## Abstract

Type 2 diabetes mellitus (T2DM) is a major global health concern with increasing prevalence and healthcare costs. Despite the availability of pharmacological interventions, sustained glycemic control and disease remission remain challenging. Dietary strategies such as low-carbohydrate diets (LCDs) and ketogenic diets (KDs) are gaining attention for their potential to improve metabolic parameters and induce T2DM remission. The objective of this review is to evaluate the long-term efficacy and safety of low-carbohydrate and ketogenic diets in the management and remission of type 2 diabetes mellitus. The systematic review was conducted following Preferred Reporting Items for Systematic Reviews and Meta-Analyses (PRISMA) guidelines. PubMed and Cochrane Library databases were searched using predefined keywords and filters. Studies included randomized and non-randomized clinical trials published in English within the last 24 years. Eligible studies involved adult T2DM patients on LCDs/KDs with a follow-up period of at least 12 months. The primary outcomes were T2DM remission, changes in glycated hemoglobin (HbA1c), body weight, body mass index (BMI), and secondary outcomes included blood pressure, lipid profiles, and adverse effects. Out of 124 initially identified studies, six studies met the inclusion criteria, with follow-up durations ranging from one to eight years. Low-carbohydrate and ketogenic diets led to significant reductions in HbA1c, body weight, BMI, and systolic blood pressure. In terms of long-term studies, remission rates were highest at one year (up to 62%) and declined to 13% by year five. Participants in the intervention groups also experienced reduced dependency on glucose-lowering and antihypertensive medications. Despite some weight regain and glycemic relapse over time, the overall metabolic improvements suggest a beneficial role of dietary intervention in T2DM management. Low-carbohydrate and ketogenic diets appear to be effective in improving metabolic outcomes and inducing remission in T2DM. These dietary interventions may serve as viable alternatives to pharmacological treatments or bariatric surgery, provided that long-term adherence and support mechanisms are in place. Further research is needed to address long-term safety, sustainability, and individualized dietary approaches.

## Introduction and background

Type 2 diabetes (T2D) is a chronic metabolic disorder characterized by elevated blood glucose levels due to insulin resistance and/or insufficient insulin production [1]. It has emerged as a global public health crisis, with the number of affected individuals rising dramatically from 200 million in 1990 to 830 million in 2022 [2]. Over 95% of diabetes cases are attributed to T2D, which, if left unmanaged, can lead to severe complications such as cardiovascular disease, neuropathy, retinopathy, and kidney failure [2]. The economic burden of T2D is staggering, with global healthcare costs estimated at $966 billion in 2021 [3]. Given its progressive nature and associated complications, effective management strategies are urgently needed to mitigate its impact on individuals and healthcare systems.

Historically, dietary interventions were the cornerstone of diabetes management before the advent of pharmacological treatments in the 1920s. Today, lifestyle modifications, including diet and physical activity, remain central to T2D management. However, the reliance on pharmacological treatments, such as oral antidiabetic drugs and insulin, has limitations, including side effects, diminishing efficacy over time, and challenges in achieving sustainable weight loss [4]. These limitations have spurred interest in alternative strategies, particularly dietary interventions, to achieve better glycemic control, reduce medication dependency, and potentially induce remission.

Among the dietary approaches gaining attention, low-carbohydrate diets (LCDs) and ketogenic diets (KDs) have shown promise in improving glycemic control, enhancing insulin sensitivity, and promoting weight loss. LCDs typically restrict carbohydrate intake to less than 130 grams per day, shifting the body’s metabolism toward fat utilization [5]. Ketogenic diets, a more restrictive form of LCDs, further reduce carbohydrate intake to induce nutritional ketosis, a metabolic state where the body uses ketones as its primary energy source [6]. Preliminary studies suggest that these diets may not only improve glycemic control but also induce remission in some individuals, defined as achieving a glycated hemoglobin (HbA1c) level below 6.5% for at least three months without glucose-lowering medications [6].

The potential for dietary interventions to induce T2D remission is particularly compelling. Bariatric surgery, a well-established method for achieving remission, is associated with significant risks, including postoperative complications occurring in 10.2% of cases within 30 days [7]. In contrast, dietary interventions such as LCDs and KDs offer a less invasive and potentially more accessible alternative. Recent studies have demonstrated that LCDs can achieve remission rates comparable to bariatric surgery, with long-term safety and efficacy [6,8,9]. For instance, the Diabetes Remission Clinical Trial (DiRECT) reported sustained remission in participants following a low-calorie, low-carbohydrate diet over five years [8]. Similarly, a continuous remote care model incorporating carbohydrate-restricted nutrition therapy demonstrated significant improvements in glycemic control and weight loss over five years [6].

Despite these promising findings, the long-term safety and efficacy of LCDs and KDs remain understudied. Concerns have been raised about potential adverse effects, including impacts on lipid profiles, kidney and liver function, and the risk of nutrient deficiencies [6,8]. Additionally, the sustainability of these diets and their effects on cardiovascular health require further investigation. For example, while some studies report improvements in cardiovascular risk factors such as blood pressure and lipid profiles, others highlight potential risks associated with increased saturated fat intake [9]. These uncertainties underscore the need for a comprehensive evaluation of the long-term impact of LCDs and KDs on T2D management.

The rationale for this review is grounded in the growing global prevalence of T2D and the limitations of current pharmacological treatments. While medications such as metformin, sulfonylureas, and insulin are effective in managing blood glucose levels, they often fail to address the underlying metabolic dysfunction and may contribute to weight gain, hypoglycemia, and other side effects [8]. In contrast, dietary interventions such as LCDs and KDs target the root causes of T2D, including insulin resistance and hyperglycemia, offering a more holistic approach to management. Furthermore, the potential for these diets to induce remission represents a paradigm shift in T2D care, moving beyond symptom management to address the disease’s underlying mechanisms.

This systematic review aims to address these gaps by synthesizing the available evidence on the long-term effects of LCDs and KDs in T2D management. Specifically, the review will evaluate the efficacy of these diets in achieving glycemic control, inducing remission, and reducing medication dependency. It will also assess their safety, focusing on metabolic markers, lipid profiles, kidney and liver function, and other potential adverse events. If LCDs and KDs are shown to be safe and effective in the long term, they could be integrated into standard care protocols as a first-line intervention for T2D. This would not only improve patient outcomes but also reduce the economic burden of diabetes on healthcare systems. By providing a critical analysis of the existing literature, this review seeks to inform clinical decision-making, guide public health policies, and identify areas for future research.

## Review

Methods

Data Source & Search Strategy

This systematic review was carried out in accordance with the recommendations provided by Preferred Reporting Items for Systematic Reviews and Meta-Analyses (PRISMA) [10,11]. A systematic database search was conducted at the first stage using the above-mentioned keywords in PubMed and Cochrane libraries. During the search, several filters were applied, including open-access studies, publication years from 2000 to 2024, the English language, and studies conducted in humans. This search, which utilized several search strings and keywords, produced a total of 850 articles. To achieve better results, several criteria were implemented, including a focus on randomized clinical trials (RCTs), non-randomized clinical trials, and cohort studies in the study design. In addition, studies that were conducted during the last twenty-four years that specifically involved human subjects and were conducted on adults were taken into consideration for inclusion in the review. In addition, only articles written in English were taken into consideration.

Inclusion and Exclusion Criteria

In this systematic review, those studies were included that met all the following inclusion criteria: (1) participants are 18 years or more, (2) diagnosed with type 2 diabetes mellitus with or without other comorbidities, (3) low-carbohydrate diets (LCDs), including ketogenic diets (KDs), with varying carbohydrate intake (≤50 g/day for KDs and 50-130 g/day for LCDs) used as intervention, (4) studies comparing LCDs/KDs with or without standard care (antidiabetic meds or other dietary interventions, e.g., low-fat diets, calorie-restricted diets) for T2D remission, (5) follow-up period at least 12 months, (6) primary outcome: achievement of T2D remission (defined by HbA1c < 6.5% or < 48 mmol/mol with or without medications for consecutive three months), weight loss, (7) secondary outcomes: lipid profiles, cardiovascular risk, kidney function, and adverse events (e.g., hypoglycemia, nutrient deficiencies), (8) study design is randomized controlled trials, non-randomized clinical trials, and (9) studies published in English and within the last 24 years having full free access.

Study Selection

The whole process of selection of studies was performed in three consecutive phases. These are the comprehensive database search and identification phase, the initial screening phase, and the full-text retrieval phase. In the preliminary database search phase, a thorough search was performed in PubMed and Cochrane libraries using specific filters. In the next step, duplicate studies were removed using EndNote and manually as well. After removal of duplication, the titles and abstracts of the articles were screened thoroughly, and inappropriate studies (those not meeting inclusion criteria) were eliminated from the list. In the next steps, the remaining studies were sought for full-text retrieval. Finally, six studies meeting all the inclusion criteria and specific filters and having full-text access were selected for the systematic review.

Data Extraction

All the selected studies were reviewed for the appropriate data extraction. The extracted data were preserved and incorporated into an Microsoft Excel sheet (Microsoft Corporation, Redmond, Washington, USA). The following data were extracted from the selected articles: first author and year, study design, sample size (intervention and control), inclusion criteria, intervention received by the intervention group, intervention received by the control group, follow-up period, and measured outcomes (baseline and post-intervention values).

Outcomes

The efficacy outcomes were evaluated as reduction of HbA1c, body weight, body mass index (BMI), systolic blood pressure, and lipid profile in this review at the end point of the treatment intervention throughout the follow-up period. Comparing the proportion of patients in the intervention and control groups allowed for the measurement of efficacy outcomes. 

Assessment of Risk Bias

For all the research studies included in this review, the risk of bias was evaluated using the JADAD score. The JADAD score [12] is a scale that is used to evaluate the methodological quality of controlled trials. Here, studies are graded on three criteria, including randomization, masking and patient accountability, and receive an overall score ranging from 0 to 5. 

Results

**Figure 1:**
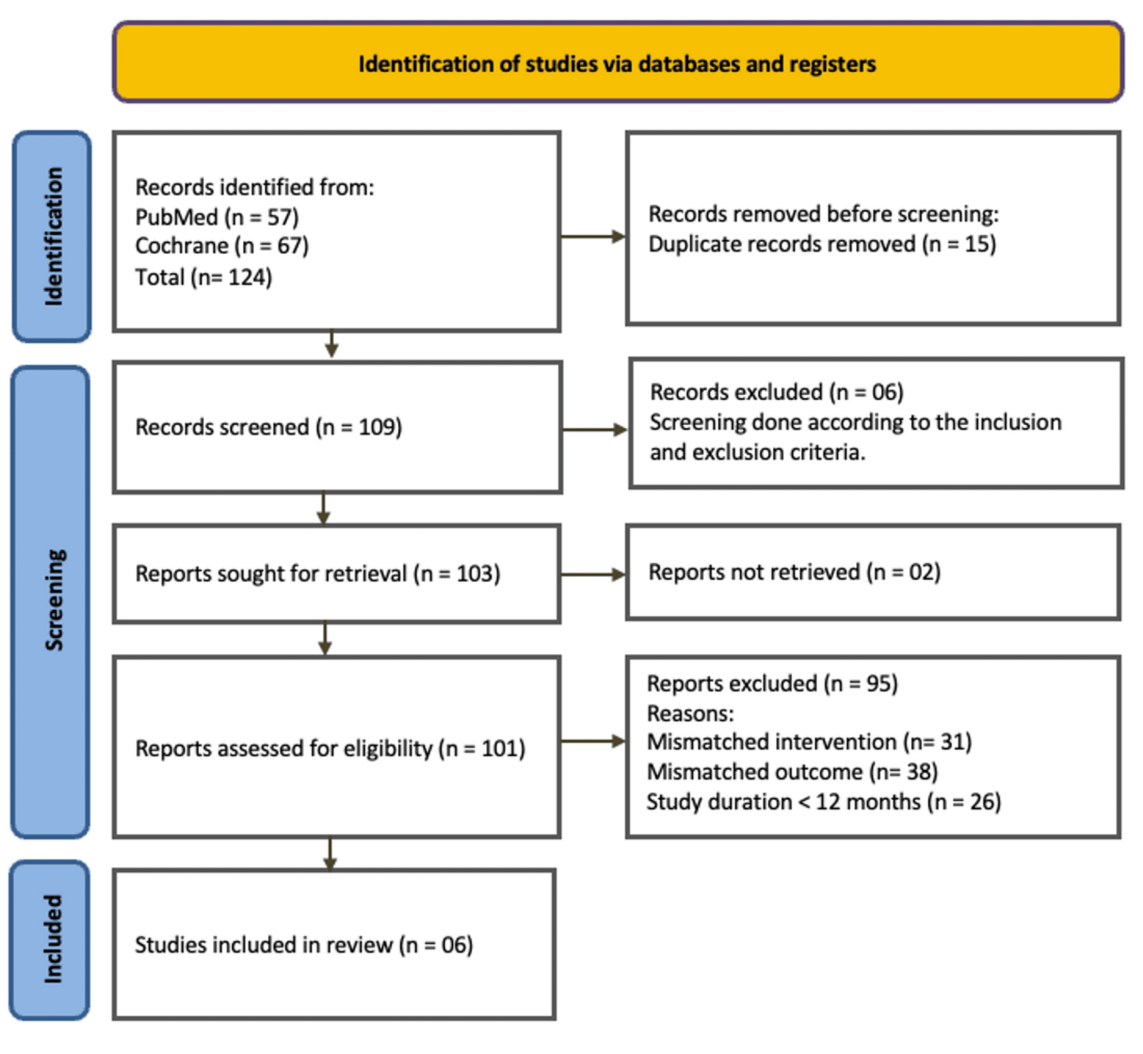
PRISMA flow diagram of study selection process. PRISMA: Preferred Reporting Items for Systematic Reviews and Meta-Analyses.

At the preliminary stage of the database search, 57 and 67 studies were found from PubMed and the Cochrane Library, respectively, using the relevant keywords. From these 124 articles, 15 duplicates were removed using EndNote. After that, the titles and abstracts of the remaining 109 articles were screened, and six of them were excluded by screening the titles and abstracts. At this stage, the remaining 103 articles were sought for full text, and two were excluded due to the unavailability of full text. From the remaining 101 articles, 95 were excluded after thorough screening of the full text for the following reasons: mismatched intervention (n = 31), mismatched outcome (n = 38), study duration <12 months (n = 26). At the last stage, after all the exclusions, six articles were selected for the review (Figure 1). PRISMA flow diagram for the selection of eligible randomized controlled trials is shown below.

The six chosen studies differed in design, duration, and dietary intervention, yet collectively aimed to assess low-carbohydrate or ketogenic diets in the management of type 2 diabetes. The majority of research utilized randomized controlled or cohort designs and involved patients with confirmed diagnoses of T2D. The durations of interventions varied from one to eight years, with differing definitions of low-carbohydrate intake (spanning from <130 g/day to <50 g/day in ketogenic protocols). Commonly evaluated outcomes encompassed HbA1c, body weight, BMI, medication utilization, blood pressure, and lipid profiles. The summative characteristics of the studies are presented in Table 1. 

**Table 1:** Characteristics of the reviewed studies. RCT: randomized controlled trial, CGM: continuous glucose monitoring, F/U: follow up.

Articles	Study design	Country	Participants (intervention/control)	Mean age (year±SD) intervention/control	Intervention	Control	Follow-up period	JADAD score
Griauzde et al. 2022 [13]	Two-arm RCT, quality improvement	USA	786/798	63.3/62.9	CGM+ low-carb diet (always hungry diet)	Usual care	12 months	3/5
Unwin et al. 2023 [9]	Single-arm clinical trial	UK	186/No control	63/NA	Low-carb diet	No control	Eight years	Not RCT
Sato et al. 2017 (one year F/U) [14]	RCT	Japan	22/27	60.5 ± 10.5/8.4 ± 10	Low-carb diet (130 g/day)	Calorie-restricted diet	18 months	2/5
Davies et al. 2009 [15]	RCT	USA	55/50	54 ± 6/53 ± 7	Low-carb diet	Low-fat diet	12 months	3/5
McKenzie et al. 2024 [6]	Cluster RCT (extension study)	UK	149/149	52.9/55.9	Total diet replacement (825-853 kcal/day) + weight maintenance support	Usual diabetes care	Five years	Not RCT
Lean et al. 2024 [8]	Non-randomized open-label extension study	USA	169/149	54.2 ± 8.3/55.9 ± 7.3	Very low-carb diet with nutritional ketosis via telemedicine	Usual care	Five years	3/5

The statistical analysis of the studies revealed significant improvements in HbA1C, body weight, BMI, systolic blood pressure, and lipid profiles among participants following low-carbohydrate diets or similar interventions. HbA1C values showed consistent reductions across studies, with Griauzde et al. (2022) reporting a decrease from 75 to 65.2 in the intervention group and Unwin et al. (2023) demonstrating a substantial reduction from 63 to 46 over an eight-year period. Similarly, Sato et al. (2017) observed a decrease from 63.9 to 56.3 in their intervention group, outperforming the control group. Body weight followed a similar trend, with Unwin et al. reporting a drop from 97 to 86 kg, Sato et al. noting a reduction from 67.9 to 66.5 kg, and McKenzie et al. (2024) achieving a significant weight loss from 116.4 to 107.6 kg. Changes in BMI were also evident, with Griauzde et al. and Sato et al. both observing reductions in their intervention groups, although control groups showed limited or no change (Table 2).

**Table 2:** Results of efficacy outcomes assessed in the reviewed studies. *Statistically significant values. HbA1C: glycated hemoglobin, BMI: body mass index.

Author, year	HbA1C (mmol/mol)				Body weight (kg)				BMI (kg/m^2^)			
	Intervention		Control		Intervention		Control		Intervention		Control	
	Baseline	Follow up	Baseline	Follow up	Baseline	Follow up	Baseline	Follow up	Baseline	Follow up	Baseline	Follow up
Griauzde et al. 2022 [13]	75	65.2*	73.8	68.6	-	-	-	-	35.84	35.17	35.45	34.89
Unwin et al. 2023 [9]	63	46*			97	86*						
Sato et al. 2017 (12 months F/U) [14]	63.9	56.3*	67.2	66.1	67.9	66.5*	73.9	73.9	26.1	25.3	26.5	26.5
Davis et al. 2009 (six months F/U) [15]	58.5	58.21	57.4	57.25	93.6	91.8*	101	96.6	-	-	-	-
													Davis et al. 2009 (12 months F/U) [15]	58.48	57.16	90.5*	97.9
McKenzie et al. 2024 [6]		58.5		55.2*		N/A		N/A										116.4	107.6*	N/A	N/A	-	-	-	-
Lean et al. 2024 [8]		60.4		50.6 (year one), 54.4 (year two), 62.2 (year three), 63.1 (year four), 66.7 (year five)		58.2		59.6 (year one), 58.6 (year two), 62.4 (year three), 64.1 (year four), 64.4 (year five)					101	*90.4 (year one), 93.2 (year two), 95.1 (year three), 93.5 (year four), 94.6 (year five)	98.8	97.7 (year one), 96.4 (year two), 94.7 (year three), 95.5 (year four), 96.4 (year five)	-	-	-	-

Systolic blood pressure exhibited significant improvements in some studies, with Unwin et al. (2023) reporting a reduction from 140 to 132 mmHg in the intervention group. While Davis et al. (2009) noted minimal changes at six months, their 12-month data indicated a reduction in systolic blood pressure in the intervention group. Improvements in lipid profiles were marked across multiple studies. Unwin et al. observed decreases in triglycerides (186 to 124 mg/dL), low-density lipoprotein (LDL) (139 to 120 mg/dL), and total cholesterol (189 to 166 mg/dL), alongside an increase in high-density lipoprotein (HDL) (43 to 46 mg/dL). Similarly, McKenzie et al. (2024) reported favorable changes, including significant reductions in triglycerides and increases in HDL. Overall, the data highlight the effectiveness of low-carbohydrate and calorie-restricted diets in improving metabolic health outcomes, particularly when implemented over the long term (Table 3). 

**Table 3:** Results of secondary outcomes assessed in the reviewed studies. *Statistically significant values. TG: triglycerides, HDL: high-density lipoprotein, LDL: low-density lipoprotein.

Author, year	Systolic blood pressure (mmHg)				Lipid profile (mg/dL)				
	Intervention		Control		Intervention			Control	
	Baseline	Follow up	Baseline	Follow up		Baseline	Follow up	Baseline	Follow up
Griauzde et al. 2022 [13]	-	-	-	-	-	-	-	-	-
Unwin et al. 2023 [9]	140	132*	-	-	TG	186	124*	-	-
					HDL	43	46*		
					LDL	139	120*		
					Cholesterol	189	166*		
Sato et al. (one year F/U) [14]	-	-	-	-	TG	128.5	134.5	162	157
					HDL	44.5	46.5	47	50
					LDL	103	93*	97	100.5
					Cholesterol	-	-	-	-
Davis et al. 2009 (six months F/U) [15]	125	124.22	130	126.3	TG	124	122.2	124	120.5
					LDL	96.7	92.8	92.8	83.12
					HDL	50.3	56.5*	46.4	46.02
					Cholesterol	170.2	173.02	166.3	155.8
Davis et al. 2009 (12 months F/U) [15]		123		128.2	TG	124	110.7	124	122.1
					LDL	96.7	95.14	92.8	85.9
					HDL	50.3	56.5*	46.4	48.7
					Cholesterol	170.2	173	166.3	161.3
McKenzie et al. 2024 [6]	-	-	-	-	TG	202.3	165.1*	-	-
					HDL	43	50.6 *	-	-
					LDL	104.6	100	-	-
					Cholesterol	182.3	179.6	-	-

Participants in the DiRECT intervention group (extension group) experienced a marked reduction in body weight during the first year of the study, decreasing from a baseline mean of 99.5 ± 16.1 kg to 87.4 ± 15.2 kg [8]. Although partial weight regain was observed in subsequent years, the average body weight at year five (93.2 ± 15.3 kg) remained significantly below baseline, indicating the long-term impact of structured dietary support. In contrast, participants in the standard care group showed minimal weight loss, highlighting the effectiveness of the intervention in achieving clinically meaningful outcomes. Glycemic control, as measured by HbA1c, also improved substantially in the intervention group. HbA1c levels dropped from 57.8 ± 10.8 mmol/mol at baseline to 46.8 ± 10.4 mmol/mol at year one. However, by year five, the mean value rose to 62.7 ± 16.1 mmol/mol, slightly surpassing baseline levels. Despite this increase, participants in the intervention group consistently spent more time with HbA1c values below 48 mmol/mol compared to controls, indicating a greater overall degree of glycemic control throughout the study period.

Remission of type 2 diabetes, defined as achieving HbA1c <48 mmol/mol without the use of glucose-lowering medications, was observed in 62% of participants in the intervention group at year one. This proportion declined steadily to 13% at year five. Despite the decrease, the sustained remission seen in a notable subset of participants underscores the potential for long-term disease reversal through lifestyle modification. Notably, remission was closely associated with weight loss of ≥10 kg, reinforcing the importance of weight management in diabetes care.

The intervention also led to substantial reductions in medication use. At baseline, most participants were prescribed glucose-lowering agents, but by year one, 87% were off such medications. Even at year five, 40% remained medication-free. A similar pattern was observed for antihypertensive drugs: the proportion of participants not requiring these medications increased from 45% at baseline to 72% at year one, before settling at 47% by year five. These findings reflect a reduced pharmacological burden and highlight potential cost savings and improved patient quality of life.

While initial improvements in blood pressure were observed, despite medication withdrawal, systolic and diastolic values returned to baseline levels (135/82 mm Hg) by year five. Nonetheless, nearly half of the participants maintained normal blood pressure without antihypertensive therapy, suggesting ongoing metabolic benefits independent of pharmacologic intervention.

Regarding lipid profiles and other biomarkers, the trial reported improvements in HDL cholesterol and temporary reductions in alanine aminotransferase (ALT), gamma-glutamyl transferase (GGT), and C-reactive protein (CRP) levels. However, total cholesterol levels increased over the five years, possibly influenced by lower statin usage in the intervention group. These findings point to a complex cardiometabolic response that may necessitate individualized lipid management strategies alongside dietary interventions.

In the reviewed studies, low-carbohydrate and very low-carbohydrate diets exhibited predominantly positive safety profiles in persons with type 2 diabetes. The five-year trial conducted by McKenzie et al. (2024) identified no significant adverse events associated with the intervention, noted improvements in liver enzymes and inflammation markers, and found no evidence of heightened cardiovascular event risk. Likewise, the DiRECT extension experiment conducted by Lean et al. (2024) revealed no significant adverse safety signals over the five-year follow-up, despite considerable medication discontinuation, indicating that structured weight reduction programs are safe in the long run. In the eight-year primary care assessment conducted by Unwin et al. (2023), no adverse events associated with carbohydrate restriction were documented, and patient feedback indicated enhancements in overall well-being. The research conducted by Griauzde et al. (2022), which incorporated continuous glucose monitoring and low-carbohydrate coaching, demonstrated substantial decreases in HbA1c without any observed rise in hypoglycemia or other negative consequences. Sato et al. (2017) randomized controlled trial indicated the absence of major adverse events over the one-year follow-up; minor gastrointestinal issues were temporary and did not result in discontinuation. Davis et al. (2009) conducted a 12-month comparison of low-carbohydrate and low-fat diets, revealing comparable safety profiles in both cohorts, with no significant differences in adverse event rates or blood pressure alterations; yet the low-carbohydrate group had a marginally larger increase in HDL cholesterol levels. These data indicate that LCDs and very low-calorie diets (VLCDs), when appropriately executed and supervised, are safe and well-accepted dietary approaches for the long-term control of T2DM. Nevertheless, personalized evaluation and clinical oversight are crucial, particularly for drug modification and patient compliance.

Discussion

This systematic review highlights how low-carbohydrate diets (LCDs) improve metabolic health & induce remission of type 2 diabetes mellitus. Analysis of the studies revealed significant improvements in HbA1C, body weight, BMI, systolic blood pressure, and lipid profiles among participants adhering to low-carbohydrate diets or similar interventions. These improvements contribute to reducing the cardiovascular risk [6,9].

LCDs have shown effectiveness in inducing weight loss, a critical factor in improving T2D management. Weight reduction enhances insulin sensitivity and lowers blood glucose levels. According to our review, HbA1C values showed consistent reductions. For instance, Griauzde et al. (2022) reported a decrease from 75 to 65.2 mmol/mol in the intervention group [13], and Unwin et al. (2023) demonstrated a substantial reduction from 63 to 46 over an eight-year period [9]. Similarly, Sato et al. (2017) observed a decrease from 63.9 to 56.3 in their intervention group, outperforming the control group [14]. These findings suggest that LCDs essentially induce diabetes remission and improve glycemic control.

Several trials confirm that weight loss is crucial for achieving remission [8]. In this systematic review, a consistent trend in weight reduction was observed, with Unwin et al. reporting a decrease from 97 to 86 kg [9], Sato et al. from 67.9 to 66.5 kg [14], and McKenzie et al. (2024) from 116.4 to 107.6 kg [6]. Additionally, reductions of BMI were also evident, with Griauzde et al. and Sato et al. both observing reductions in their intervention groups, although control groups showed limited or no change [13,14]. 

Multiple research projects consistently show that reductions in pancreatic and hepatic fat improve beta-cell function and insulin sensitivity. Conversely, fat re-accumulation and diabetes relapse were linked to weight regain, emphasizing long-term weight maintenance techniques [16-18]. Metabolic age is more significant than chronological age, as a shorter duration of diabetes has a better success rate [8,9]. 

Notably, studies of low-carbohydrate diets with concomitant calorie restriction (e.g., max 800 kcal/day) leading to substantial weight loss report much higher rates of diabetes remission than other studies of low-carbohydrate diets without calorie restriction, even when weight loss is similar in both the intervention and control groups [19,20].

Two other narrative reviews (without documented search strategies) concluded that both low-carbohydrate diets and energy-restricted diets are effective in managing T2D [21,22]. A 2022 narrative review also highlighted that maintaining weight loss is the primary determinant of sustaining the effects of a low-carbohydrate diet on diabetes remission [21]. Moreover, there are concerns that blood glucose levels could potentially rise again once more carbohydrates are reintroduced or if weight loss is not maintained. In other words, the glycemic effects of low-carbohydrate diets may constitute diabetes mitigation and not physiological remission because the underlying pathology may not be altered in the absence of sufficient weight loss. True physiological remission is likely only achievable when fat around the organs is lost, which enables insulin production and utilization [21]. However, a major limitation of current literature is the absence of study designs of sufficient length for measuring diabetes remission to manifest as a primary outcome.

It is important to adhere to this intervention and control support for sustained improvement to ensure the persistent enhancement of health parameters. Due to carbohydrate craving, it is challenging to adhere to a diet, as it exhibits characteristics akin to addiction [23,24]. This emphasizes the necessity for ongoing support to maintain consistency and improvement of metabolic health [8,25]. Future work should be done to establish a clear threshold for carbohydrate restriction and duration of adherence that optimizes the clinical outcomes. For long-term compliance and continuous encouragement and supportive contact, even a telephone call proves to be beneficial [9].

The composition of carbohydrates-whether derived from whole grains, vegetables, or processed foods-has a significant impact on the efficacy of a dietary regimen. LCDs that focus on nutrient-dense, low-glycemic foods may offer superior outcomes than those high in added sugars or refined carbohydrates. Both low-fat diets and LCDs have demonstrated improvements in weight reduction. However, LCDs showed better glycemic control and HbA1c improvement [14,26].

The Look AHEAD study [27] indicated that the conventional meal replacements and increased physical activity achieved only 7.3% remission over four years. In contrast, the DiRECT trial [8], which utilized a low-calorie diet replacement strategy, reported a 13% remission rate over five years. The Mediterranean diet showed a probability of remission from metabolic syndrome of 49% [28], though it targeted metabolic syndrome more broadly, not specifically type 2 diabetes remission. On the other hand, LCDs can induce remission as high as 51% [6,9]. Additional research indicates a reduction in inflammatory markers like high-sensitivity C-reactive protein (hsCRP), while dietary ketosis contributes to further metabolic health improvement [29-31].

Significant weight reduction may lead to a transient elevation in cholesterol levels; however, this has no adverse long-term consequences. Besides improvement in glycemic management and type 2 diabetes remission, such dietary approaches have been linked to a decreased risk of cardiovascular and chronic kidney diseases [32,33]. Further highlighting that dietary modification in diabetes care can facilitate temporary remission with protective benefits against both macro- and microvascular complications [32]. Despite these promising findings, certain limitations must be acknowledged, for example, self-reported dietary compliance and the absence of long-term randomized control trials. Some studies have documented a decline in efficacy after 12 months, probably due to poor compliance [19,26]. By assessing safety, efficacy, and long-term compliance with this dietary intervention across a diverse population, future research could address and close this gap. 

The intervention's safety outcomes indicate that low- and very low-carbohydrate diets are safe and well-tolerated during the follow-up period. No significant adverse events or fatalities were observed during the follow-up period. A systematic review and meta-analysis published in The BMJ (2021), comprising 23 randomized controlled trials with 1357 participants, assessed the efficacy and safety of low- and very low-carbohydrate diets (LCDs/VLCDs) in persons with type 2 diabetes. The review identified no substantial rise in adverse events among participants adhering to LCDs or VLCDs in comparison to control diets over a duration of six to 12 months [19]. Furthermore, although quality of life assessments indicated no difference at six months, there was a clinically significant, if not statistically significant, deterioration at 12 months in the low-carb groups. Triglyceride readings improved; however, LDL cholesterol exhibited a non-significant rise at 12 months. These data indicate that LCDs and VLCDs are typically safe and well-tolerated in the short term, providing metabolic advantages without significant adverse effects. The evaluation underscores the necessity for additional research about their long-term safety and sustainability in standard diabetic management.

Considering all aspects, a low-carbohydrate diet represents a pragmatic and successful dietary strategy for type 2 diabetes remission, particularly when combined with ongoing weight management and a support system. Dietary recommendations should be customized according to personal preferences, metabolic profile, and the potential for long-term adherence.

This review has multiple limitations to be considered. The number of high-quality long-term randomized controlled trials evaluating low-carbohydrate or ketogenic diets for type 2 diabetes remission is limited, hence constraining the strength of the evidence base. Secondly, variability in study design, dietary treatments, and follow-up lengths hinders direct comparison and prevents meta-analytic synthesis. Third, the majority of research depended on self-reported dietary adherence, potentially introducing reporting bias and compromising outcome reliability. Fourth, there was insufficient data regarding adverse events, cardiovascular outcomes, and long-term durability, which are essential for evaluating clinical applicability. The lack of individual participant data limited subgroup analyses, including stratification by age, disease duration, or baseline metabolic condition, which could yield more detailed insights into dietary efficacy. For future research, the following aspects should be given more focus to obtain more robust outcomes: stratification of results by age and other demographic factors; direct comparisons between low-carbohydrate/ketogenic diets and alternative dietary approaches (e.g., plant-based, intermittent fasting); and studies evaluating potential synergistic effects when combining different dietary strategies.

## Conclusions

This systematic review underscores the efficacy of low-carbohydrate and ketogenic diets as viable strategies for treating and attaining remission in type 2 diabetes mellitus with the available study data. Long-term trial evidence indicates significant enhancements in glycemic control, body weight, and cardiometabolic health, with certain individuals maintaining remission for multiple years. These findings highlight the significance of dietary treatments as effective supplements or potential substitutes to traditional pharmacological or surgical methods. Nonetheless, obstacles persist in sustaining long-term compliance and providing personalized assistance. Future studies must concentrate on refining dietary guidelines, pinpointing patient subgroups with the highest potential for benefit, and assessing the overall effects on quality of life and long-term safety. Incorporating systematic dietary interventions into basic care could profoundly transform diabetes management and provide renewed optimism for disease reversal.

## References

[1] (2025). Diabetes UK. Know diabetes. Fight diabetes. https://www.diabetes.org.uk..

[2] (2025). World Health Organization. https://www.who.int..

[3] International Diabetes Federation (2025). International Diabetes Federation. IDF Diabetes Atlas. https://diabetesatlas.org.

[4] Scheen AJ (2017). Pharmacotherapy of 'treatment resistant' type 2 diabetes. Expert Opin Pharmacother.

[5] Galicia-Garcia U, Benito-Vicente A, Jebari S (2020). Pathophysiology of type 2 diabetes mellitus. Int J Mol Sci.

[6] McKenzie AL, Athinarayanan SJ, Van Tieghem MR (2024). 5-Year effects of a novel continuous remote care model with carbohydrate-restricted nutrition therapy including nutritional ketosis in type 2 diabetes: an extension study. Diabetes Res Clin Pract.

[7] Jans A, Näslund I, Ottosson J, Szabo E, Näslund E, Stenberg E (2019). Duration of type 2 diabetes and remission rates after bariatric surgery in Sweden 2007-2015: a registry-based cohort study. PLoS Med.

[8] Lean ME, Leslie WS, Barnes AC (2024). 5-year follow-up of the randomised Diabetes Remission Clinical Trial (DiRECT) of continued support for weight loss maintenance in the UK: an extension study. Lancet Diabetes Endocrinol.

[9] Unwin D, Delon C, Unwin J, Tobin S, Taylor R (2023). What predicts drug-free type 2 diabetes remission? Insights from an 8-year general practice service evaluation of a lower carbohydrate diet with weight loss. BMJ Nutr Prev Health.

[10] Sarkis-Onofre R, Catalá-López F, Aromataris E, Lockwood C (2021). How to properly use the PRISMA statement. Syst Rev.

[11] Page MJ, McKenzie JE, Bossuyt PM (2021). The PRISMA 2020 statement: an updated guideline for reporting systematic reviews. Int J Surg.

[12] Jadad AR, Moore RA, Carroll D (1996). Assessing the quality of reports of randomized clinical trials: is blinding necessary?. Control Clin Trials.

[13] Griauzde DH, Ling G, Wray D (2022). Continuous glucose monitoring with low-carbohydrate nutritional coaching to improve type 2 diabetes control: randomized quality improvement program. J Med Internet Res.

[14] Sato J, Kanazawa A, Hatae C (2017). One year follow-up after a randomized controlled trial of a 130 g/day low-carbohydrate diet in patients with type 2 diabetes mellitus and poor glycemic control. PLoS One.

[15] Davis NJ, Tomuta N, Schechter C (2009). Comparative study of the effects of a 1-year dietary intervention of a low-carbohydrate diet versus a low-fat diet on weight and glycemic control in type 2 diabetes. Diabetes Care.

[16] Lu X, Xie Q, Pan X (2024). Type 2 diabetes mellitus in adults: pathogenesis, prevention and therapy. Signal Transduct Target Ther.

[17] Taylor R (2013). Banting memorial lecture 2012: reversing the twin cycles of type 2 diabetes. Diabet Med.

[18] Al-Mrabeh A, Zhyzhneuskaya SV, Peters C (2020). Hepatic lipoprotein export and remission of human type 2 diabetes after weight loss. Cell Metab.

[19] Goldenberg JZ, Day A, Brinkworth GD (2021). Efficacy and safety of low and very low carbohydrate diets for type 2 diabetes remission: systematic review and meta-analysis of published and unpublished randomized trial data. BMJ.

[20] Athinarayanan SJ, Roberts CG, Vangala C, Shetty GK, McKenzie AL, Weimbs T, Volek JS (2024). The case for a ketogenic diet in the management of kidney disease. BMJ Open Diabetes Res Care.

[21] Brown A, McArdle P, Taplin J (2022). Dietary strategies for remission of type 2 diabetes: a narrative review. J Hum Nutr Diet.

[22] Arias-Marroquín AT, Del Razo-Olvera FM, Castañeda-Bernal ZM (2024). Personalized versus non-personalized nutritional recommendations/interventions for type 2 diabetes mellitus remission: a narrative review. Diabetes Ther.

[23] Gordon EL, Ariel-Donges AH, Bauman V, Merlo LJ (2018). What is the evidence for “food addiction?” A systematic review. Nutrients.

[24] Schulte EM, Avena NM, Gearhardt AN (2015). Which foods may be addictive? The roles of processing, fat content, and glycemic load. PLoS One.

[25] Athinarayanan SJ, Adams RN, Hallberg SJ (2019). Long-term effects of a novel continuous remote care intervention including nutritional ketosis for the management of type 2 diabetes: a 2-year non-randomized clinical trial. Front Endocrinol (Lausanne).

[26] Nicholas AP, Soto-Mota A, Lambert H, Collins AL (2021). Restricting carbohydrates and calories in the treatment of type 2 diabetes: a systematic review of the effectiveness of 'low-carbohydrate' interventions with differing energy levels. J Nutr Sci.

[27] Wadden TA, West DS, Delahanty L (2006). The Look AHEAD study: a description of the lifestyle intervention and the evidence supporting it. Obesity (Silver Spring).

[28] Esposito K, Maiorino MI, Bellastella G, Chiodini P, Panagiotakos D, Giugliano D (2015). A journey into a Mediterranean diet and type 2 diabetes: a systematic review with meta-analyses. BMJ Open.

[29] Newman JC, Verdin E (2017). β-Hydroxybutyrate: a signaling metabolite. Annu Rev Nutr.

[30] Rondanelli M, Gasparri C, Pirola M, Barrile GC, Moroni A, Sajoux I, Perna S (2024). Does the ketogenic diet mediate inflammation markers in obese and overweight adults? A systematic review and meta-analysis of randomized clinical trials. Nutrients.

[31] Ji J, Fotros D, Sohouli MH, Velu P, Fatahi S, Liu Y (2025). The effect of a ketogenic diet on inflammation-related markers: a systematic review and meta-analysis of randomized controlled trials. Nutr Rev.

[32] Gregg EW, Chen H, Bancks MP, Manalac R, Maruthur N, Munshi M, Wing R (2024). Impact of remission from type 2 diabetes on long-term health outcomes: findings from the Look AHEAD study. Diabetologia.

[33] Dambha-Miller H, Day A, Kinmonth AL, Griffin SJ (2021). Primary care experience and remission of type 2 diabetes: a population-based prospective cohort study. Fam Pract.

